# Bacterial profile and antimicrobial susceptibility patterns in chronic suppurative otitis media at the University of Gondar Comprehensive Specialized Hospital, Northwest Ethiopia

**DOI:** 10.1186/s13104-019-4452-4

**Published:** 2019-07-15

**Authors:** Rahel Molla, Moges Tiruneh, Wondwossen Abebe, Feleke Moges

**Affiliations:** 1 Department of Laboratory, University of Gondar Comprehensive Specialized Hospital, Gondar, Ethiopia; 2 Department of Medical Microbiology, School of Biomedical and Laboratory Sciences, College of Medicine and Health Sciences, University of Gondar, P.O. Box: 196, Gondar, Ethiopia

**Keywords:** Chronic suppurative otitis media, Antimicrobial susceptibility patterns

## Abstract

**Objectives:**

This study aims to determine bacterial profile and antimicrobial susceptibility patterns of chronic suppurative otitis media in the University of Gondar Comprehensive Specialized Hospital, Northwest Ethiopia.

**Result:**

Sixty-two ear swabs were collected and 74 bacterial isolates were identified, of which 48 (77.4%) sample with mono-microbial growth, 11 (17.8%) with polymicrobial growth and the remaining 3 (4.8%) show no growth. The most common isolates were *Proteus mirabilis* 16 (21.6%), followed by *S. aureus* 12 (16.2%), *Klebsiella* spp. 10 (13.5%) and *Providencia* spp. 11 (14.9%). *Proteus mirabilis* was 100% susceptible to norfloxacin and ciprofloxacin while 87.5% of the isolates were susceptible to cefixime and gentamicin. *S. aureus* was 83.3% susceptible to gentamicin and clarithromycin, while 75% of the isolates were susceptible to amoxicillin–clavulanic acid and chloramphenicol, however, 66.7% the isolates were susceptible to ciprofloxacin, norfloxacin and erythromycin. The overall prevalence of multidrug resistance in the current study was 35 (47.3%). In this study *P. mirabilis*, *S. aureus*, *Providencia* spp., and *Klebsiella* spp. were the most common bacterial isolate and all Gram negative isolates were susceptible to ciprofloxacin and norfloxacin. Amoxicillin–clavulanic acid, gentamicin, chloramphenicol, clarithromycin and tobramycin were relatively effective against Gram positive bacteria.

**Electronic supplementary material:**

The online version of this article (10.1186/s13104-019-4452-4) contains supplementary material, which is available to authorized users.

## Introduction

Chronic suppurative otitis media (CSOM) is defined as a persistent infection of the middle ear with a perforated tympanic membrane draining exudate for more than 6 weeks and is often associated with cholesteatoma [1, 2]. Every year, approximately 31 million people developed CSOM around the world. A lot of variation is observed in the incidence of CSOM globally, developed countries have a quite low prevalence, whereas CSOM is more prevalent in developing countries and the burden may be three times larger than developed countries [2]. Moreover, due to the high prevalence in developing countries (including Ethiopia), the World Health Organization (WHO) has categorized CSOM as neglected tropical diseases [3].

In most cases, CSOM occurs in the first 6 years of childhood, but can persist during adulthood following poor management of acute otitis media. However, accurate diagnosis of CSOM remains a difficult task due to heavily debate the exact point in time as to when AOM becomes CSOM [1, 3].

The etiology and antimicrobial resistance patterns of CSOM infection are different in different geographical area and population studied. CSOM is predominantly caused by *Pseudomonas aeruginosa*, *Escherichia coli*, *S. aureus*, *Streptococcus pyogenes*, *Proteus mirabilis*, *Klebsiella* species among aerobic bacteria. However, *Bacteroides*, *Peptostreptococcus*, *Propionibacterium* are common anaerobic bacteria [1, 4].

There are few studies documented in Ethiopia, for instance a study carried out in Addis Ababa revealed a high prevalence, 52.8% COSM among otitis media patients and the most common isolates were *Klebsiella* spp., *E. coli*, and *Diphtheroids* with the prevalence of 28.97%, 10.7%, and 7.3%, respectively [5]. Another similar study conducted in Dessie showed that the prevalence of COSM was 83.2% and the predominant isolates were *Proteus* spp., *S. aureus* and *Pseudomonas* spp. with a prevalence of 23.2%, 21%, and 14.5%, respectively [6]. Thus, having current information on the etiologies responsible for COSM and their antimicrobial susceptibility pattern is an important for prompt and effective treatment. Therefore, the aim of this study was to determine bacterial profile and their antimicrobial susceptibility patterns in COSM from patients attending the University of Gondar Comprehensive Specialized Hospital, Northwest Ethiopia.

## Main text

### Methods

#### Study design, area and population

A hospital-based cross-sectional study was conducted from January to June 2017 at the University of Gondar Comprehensive Specialized Hospital. University of Gondar Comprehensive Specialized Hospital is one of the largest comprehensive, specialized hospitals served as a teaching as well as patient-care in Amhara region. It is located in Gondar town, 750 km far from Addis Ababa in Northwest Ethiopia. The hospital provides surgical, medical, pediatric, gynecologic and obstetrics, intensive care unit and ear, nose and throat (ENT) clinic to the community. The hospital has an accredited laboratory, more than 1200 beds and provides health care referral services for more than 5 million people from the surrounding zones and nearby regions. The ENT clinic gives service for about 2640 patients per year.

#### Sample size and sampling technique

The sample size was calculated based on the assumption of 5% expected margins of error and 95% confidence interval, taking the prevalence of 4.2% from the previous study, which was conducted by WHO surveys studies [1] using a single population proportion formula as follows.

$${\text{n}} = \frac{{\left( {{\text{Z}}\upalpha/2} \right)^{2} {\text{P}}\left( {1 - {\text{P}}} \right)}}{{{\text{d}}^{2} }}$$

where n is the calculated sample size; Z is the standard normal deviate at 95%, confidence interval = 1.96; P is the prevalence from the previous study = 4.2%; d is the precision level = 0.05.

The total calculated sample size was 62 study participants. The study participants were enrolled consecutively using a convenience sampling technique until a sample size of 62 study participants was achieved. All study participants had perforated tympanic membranes with active purulent discharge. The detailed information regarding age, sex, duration of discharge, and the antibiotic is taken prior to data collection was collected from each study participant using a structured questionnaire by the attending ENT specialist. Whereas discharge of less than 6 weeks duration, discharge with intact tympanic membrane (otitis externa), and patient receiving antibiotic therapy (topical or systemic) within 7 days before data collection were excluded.

#### Culture and identification

The middle-ear discharge was collected by an ENT specialist under strict aseptic conditions using single-use mini-tip culture swabs, after cleaning the external auditory canal with a spirit swab. The swabs were transported to the bacteriology laboratory in the biomedical complex at the School of Biomedical and Laboratory Sciences for culture and susceptibility testing. The swab was directly inoculated on 5% sheep blood agar, chocolate agar, and MacConkey agar (HiMedia, India). The blood and MacConkey agar plates were incubated aerobically while chocolate agar was incubated under 5% CO_2_ atmosphere at 37 °C for 24–48 h. The isolates were identified by colony morphology, Gram stain, oxidase test, triple sugar iron agar, indole production, H_2_S production, citrate utilization, motility test, urease test, carbohydrate utilization tests, catalase, coagulase, DNase, bacitracin, and optochin susceptibility tests [7].

Antimicrobial susceptibility tests were performed using a modified Kirby–Bauer disc diffusion method following the Clinical Laboratory Standards Institute guidelines (2017 edition) [8]. Gram-positive isolates were tested against gentamicin (10 µg), ciprofloxacin (5 µg), trimethoprim/sulphamethoxazole (co-trimoxazole) (1.25/23.75 µg), chloramphenicol (30 µg), amoxicillin–clavulanic acid (20 µg), doxycycline (30 µg), tobramycin (10 µg), norfloxacin (10 µg), penicillin (10 µg), clarithromycin (15 µg), and erythromycin (15 µg). Gram-negative isolates were tested against gentamicin (10 µg), ciprofloxacin (5 µg), co-trimoxazole (1.25/23.75 µg), chloramphenicol (30 µg), amoxicillin–clavulanic acid (20 µg), ampicillin (10 µg), cefepime (30 µg), cefuroxime (30 µg), cefixime (5 µg), tobramycin (10 µg), tetracycline (30 µg), nalidixic acid (30 µg), and norfloxacin (10 µg) (all are routinely and locally used antibiotics from Oxoid Limited). Inoculums were prepared using 0.5 McFarland standard and inoculated on Mueller–Hinton agar (Oxoid Limited); the antibiotic disc was dispensed after drying the plate for 3–5 min and incubated at 37 °C for 24 h [8]. In this study, intermediate susceptibility to antimicrobial agents was categorized as resistance in the data analysis. Multiple drug resistance is defined as the resistance of an isolate to three and more antimicrobial agents within one class of drug [9]. The reference strains used as control were *S. aureus* (ATCC 25923), *Escherichia coli* (ATCC 25922), and *P. aeruginosa* (ATCC 27853).

#### Data analysis and interpretation

Data were entered and analyzed using SPSS version 20 software. Results were presented through graphs and tables. The statistical significance of association was measured by using the Chi-square test. A p-value < 0.05 was considered as statistically significant.

### Results

#### Socio-demographic characteristics

A total of 62 patients who had CSOM were enrolled. Thirty (48.4%) were female and 32 (51.6%) were male. The mean age of the study participant was 25 years ranged from 1 to 74 years. Twenty (32.3%) of them were aged below 15 years and 27 (43.5%) were between 16 and 30 years. Thirty-eight (61.3%) of the patients were from the urban area, 42 (67.7%) ear discharge was from adults. Fifty-nine (95.2%) of the patients had unilateral CSOM, involving the right ear in 33 (53.2%) (Additional file 1: Table S1).

#### Prevalence of bacterial isolates

**Fig. 1:**
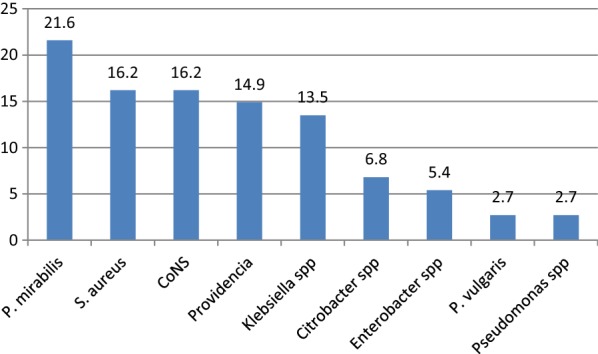
Frequencies of bacterial isolates in CSOM at the University of Gondar Comprehensive Specialized Hospital, Northwest Ethiopia from January to May 2017

Of the 62 ear swabs, 74 bacterial isolates were identified. Of which 48 (77.4%) sample with mono-microbial growth, 11 (17.8%) with polymicrobial growth and the remaining 3 (4.8%) show no growth. Among 74 isolates, 24 (32.4%) were Gram-positive bacteria, while 50 (67.6%) were Gram-negative bacteria. The most common isolates were *P. mirabilis* (16; 21.6%), followed by *S. aureus* (12; 16.2%), coagulase negative *Staphylococci* (CoNS) (12; 16.2%), *Providencia* spp. (11; 14.9%), *Klebsiella* spp. (10; 13.5%), *Citrobacter* spp. (5; 6.8%), *Enterobacter* spp. (4; 5.4%), *P. vulgaris* (2; 2.7%), and *Pseudomonas* spp. (2; 2.7%) (Fig. 1).

#### Antimicrobial susceptibility patterns

Tables 1 and 2 describe antimicrobial susceptibility patterns of most commonly isolated Gram-negative and Gram-positive bacteria, respectively. *Proteus mirabilis* was 100% susceptible to norfloxacin and ciprofloxacin, while 87.5%, of the isolates were susceptible to cefixime and gentamicin, and nalidixic acid (81.3%). *Klebsiella* spp. was susceptible to norfloxacin (100%), ciprofloxacin (100%), cefixime (80%), gentamicin (90%) and nalidixic acid (90%). *Providencia* spp. showed 100% susceptible to norfloxacin and ciprofloxacin.

**Table 1:** Antimicrobial resistance patterns of Gram negative bacteria isolated in CSOM patients at the University of Gondar Comprehensive Specialized Hospital, January–May 2017

Isolates	Antimicrobial resistance pattern											
	CFP n (%)	COT n (%)	CXM n (%)	NOR n (%)	CAF n (%)	CIP n (%)	NAL n (%)	AMP n (%)	GEN n (%)	TE n (%)	AMO/C n (%)	CFR n (%)
*P. mirabilis* (n = 16)												
R	7 (43.7)	8 (50)	2 (12.5)	0	6 (37.5)	0	3 (18.7)	13 (81.2)	2 (12.5)	9 (56.3)	5 (31.3)	5 (31.3)
*Providencia* spp. (n = 11)												
R	9 (81.8)	9 (81.8)	11 (100)	0	8 (72.7)	0	8 (72.7)	9 (81.8)	3 (27.3)	7 (63.7)	10 (90.9)	10 (90.9)
*Klebsiella* spp. (n = 10)												
R	5 (50)	4 (40)	2 (20)	0	1 (10)	0	1 (10)	9 (90)	1 (10)	1 (10)	6 (60)	5 (50)
*Citrobacter* spp. (n = 5)												
R	1 (20)	0	2 (40)	1 (20)	1 (20)	1 (20)	0	5 (100)	0	1 (20)	0	5 (100)
*Enterobacter* spp. (n = 4)												
R	2 (50)	0	2 (50)	0	0	0	0	100	0	0	0	4 (100)
*Pseudomonas* spp. (n = 2)												
R	2 (100)	2 (100)	2 (100)	0	1 (50)	0	0	0	1 (50)	2 (100)	2 (100)	2 (100)
*P. vulgaris* (n = 2)												
R	1 (50)	2 (100)	0	1 (50)	1 (50)	2 (100)	2 (100)	1 (50)	1 (50)	2 (100)	2 (100)	1 (50)

*CFP* cefepime, *COT* cotrimoxazol, *CXM* cefixime, *NOR* norfloxacin, *GEN* gentamicin, *AMO/C* amoxicillin–clavulanic acid, *CAF* chloramphenicol, *CIP* ciprofloxacin, *TE* tetracyclin, *CFR* cefuroxime, *NA* nalidixic acid, *AMP* ampicillin, *R* resistance

Tobramycin and piperacillin were replaced ampicillin and nalidixic acid respectively in case of *P. aeruginosa*

**Table 2:** Antimicrobial resistance patterns of Gram positive bacteria isolated in CSOM patients at the University of Gondar Comprehensive Specialized Hospital, January–May 2017

Isolates	Antimicrobial resistance pattern										
	GEN n (%)	AMO/C n (%)	CAF n (%)	CIP n (%)	COT n (%)	NOR n (%)	DOX n (%)	CLM n (%)	E n (%)	PEN n (%)	TOB n (%)
*S. aureus* (n = 12)											
R	2 (16.7)	3 (25)	3 (25)	4 (33.3)	6 (50)	4 (33.3)	5 (41.6)	2 (16.7)	4 (33.3)	12 (100)	3 (25)
*CoNS* (n = 12)											
R	0	0	2 (16.7)	3 (25)	10 (83.3)	3 (25)	3 (25)	4 (33.3)	5 (41.7)	9 (75)	2 (16.7)

*CoNS* coagulase negative staphylococci, *GEN* gentamicin, *AMO/C* amoxicillin–clavulanic acid, *CAF* chloramphenicol, *CIP* ciprofloxacin, *COT* cotrimoxazol, *NOR* norfloxacin, *DOX* doxycycline, *CLM* clarithromycin, *E* erythromycin, *PEN* penicillin, *TOB* tobramycin, *R* resistance

Among Gram-positive bacteria, *S. aureus* was susceptible to gentamicin (83.3%), clarithromycin (83.3%), amoxicillin–clavulanic (75%) acid, chloramphenicol (75%), ciprofloxacin (66.7%) and erythromycin (66.7%). Coagulase-negative *Staphylococcus* showed 100% susceptibility to gentamicin and amoxicillin–clavulanic acid. The higher number of multi-drug resistance (MDR) isolates were recorded in *Providencia* spp. 10 (90.9%). The overall MDR resistance in this study was 35 (47.3%) (Additional file 2: Table S2).

### Discussion

The present study provides information on the distribution of bacterial isolates causing chronic suppurative otitis media along with their antibiotic susceptibility pattern that plays a decisive role in effective management of the cases. The overall bacterial isolates in this study (95.2%) was in agreement with the study conducted in Dessie, Ethiopia (91.7%) [10], Nigeria (94.7%) [11], Kenya (95.4%) [12], Malawi (98.3%) [13], Singapore (97.8%) [14] and Iran (97.3%) [15]. However, higher than the reported prevalence by other studies in Hawassa, Ethiopia (30.8%) [16] and Addis Ababa, Ethiopia (48.5%) [17]. Gram-negative bacteria (67.6%) were the predominant isolates than Gram-positive bacteria (32.4%). This was comparable with the previous study done in Dessie, Ethiopia (78.2%) [6], Jimma, Ethiopia (75.6%) [18], Addis Ababa, Ethiopia (69.6%) [19], Nigeria (76.3%) [11], Malawi (72.4%) [13] and Iraq (62.5%) [20]. The observed differences in rates of bacterial isolation could be attributed to differences in population characteristics, variation in climate [6, 13, 18] and relatively small in terms of sample size and anaerobic cultures was not performed in this study.

In this study, the most repeatedly detected bacterial isolates were *P. mirabilis* and *S. aureus.* This finding was in line with a study conducted in Ethiopia [19, 21, 22] and other African countries like Kenya [12] and Malawi [13] where *P. mirabilis* and *S. aureus* were dominant isolates. In contrast to this study, several similar studies [23–25] in different countries reported that *P. aeruginosa* was the predominant isolates. In addition, following the predominant bacteria, *Providencia* spp, *Klebsiella* spp., *Citrobacter* spp., *Enterobacter* spp., *P. vulgaris* and *Pseudomonas* were also documented in this study. This variation might be due to small sample size and anaerobic cultures not performed in present study, geographical variation, study population, duration of ear discharge, indiscriminate use of antibiotics and cultural difference. In addition, instillation of holy oil (local residents called Kibakidus) and holy water in ear canal is the most common practicing habit as the traditional medicine in the society.

In this study, 48 (77.4%) samples were identified as mono-microbial growth, 11 (17.8%) were polymicrobial growth and the remaining 3 (4.8%) show no growth. This observation was supported by other studies [6, 10, 13]. CSOM can be characterized by co-infections with polymicrobial growth.

This study also provides insights into the susceptibility profile of bacteria isolated from ear infections. In general, our result has demonstrated that ciprofloxacin, norfloxacin, nalidixic acid, gentamicin and chloramphenicol are effective against Gram-negative bacteria and cephalosporin family, such as cefixime, cefuroxime, cefepime showed a variable range of susceptibility against Gram-negative bacteria. However, amoxicillin–clavulanic acid, gentamicin, chloramphenicol, clarithromycin, and tobramycin were relatively active against Gram-positive bacteria. Antimicrobial resistance patterns are influenced by source of the isolates, classes of antimicrobial agents, pressure exerted by antimicrobial use, and geographic location [26].

A high number of multi-drug resistance isolates was documented in *Providencia* spp. (90.9%). This may due to, *Providencia* spp. are capable of producing inducible β-lactamases that will hydrolyze primary and extended-spectrum penicillins and cephalosporins [27]. The overall MDR in this study was 35 (47.3%). This study is lower than a study conducted in Hawassa (74.5%) [16]. This variation might be due to a difference in definition of multi-drug resistance between a study period. In the previous studies MDR define as resistance to two or more classes of antimicrobials.

### Conclusions

*Proteus mirabilis*, *S. aureus*, *Providencia* spp., and *Klebsiella* spp. were the principal bacterial isolate responsible for causing CSOM in the study area. Among the tested antimicrobials ciprofloxacin, norfloxacin, nalidixic acid, gentamicin, and chloramphenicol were effective against Gram-negative bacteria. Additionally, cephalosporin family, such as cefixime, cefuroxime, cefepime showed a variable range of susceptibility against Gram-negative bacteria. However, amoxicillin–clavulanic acid, gentamicin, chloramphenicol, clarithromycin, and tobramycin were relatively effective against Gram-positive bacteria.

## Limitation of the study

The study has some limitation, relatively small in terms of sample size and anaerobic cultures not performed.

## Additional files

**Additional file 1: Table S1.** Socio-demographic characteristics of CSOM patients at the University of Gondar Comprehensive Specialized Hospital from January to May 2017.

**Additional file 2: Table S2.** Multidrug resistance patterns of the bacterial isolated in CSOM patients at the University of Gondar Comprehensive Specialized Hospital, January to May 2017.

## Data Availability

All data generated or analyzed during this study were included in this article.

## Abbreviations

CoNS: coagulase negative *Staphylococci*
CSOM: chronic suppurative otitis media
ENT: ear, nose and throat
MDR: multidrug resistance

## References

[1] Acuin Jose, World Health Organization. Dept. of Child and Adolescent Health and Development & WHO Programme for the Prevention of Blindness and Deafness. Chronic suppurative otitis media: burden of illness and management options. Geneve: World Health Organization; 2004. http://www.who.int/iris/handle/10665/42941.

[2] Monasta L, Ronfani L, Marchetti F, Montico M, Brumatti LV, Bavcar A, Grasso D, Barbiero C, Tamburlini G (2012). Burden of disease caused by otitis media: systematic review and global estimates. PLoS ONE.

[3] Li MG, Hotez PJ, Vrabec JT, Donovan DT (2015). Is chronic suppurative otitis media a neglected tropical disease?. PLoS Negl Trop Dis.

[4] Mittal R, Lisi CV, Gerring R, Mittal J, Mathee K, Narasimhan G, Azad RK, Yao Q, Grati MH, Yan D, Eshraghi AA (2015). Current concepts in the pathogenesis and treatment of chronic suppurative otitis media. J Med Microbiol.

[5] Tessema G (2001). Otitis media seen in Yekatit 12 Hospital. Ethiop Med J.

[6] Seid A, Deribe F, Ali K, Kibru G (2013). Bacterial otitis media in all age group of patients seen at Dessie referral hospital, North East Ethiopia. Egypt J Ear Nose Throat Allied Sci.

[7] Cheesbrough M (2006). District laboratory practice in tropical countries.

[8] CLSI (2017). Performance Standards for Antimicrobial Susceptibility Testing.

[9] Magiorakos AP, Srinivasan A, Carey RB, Carmeli Y, Falagas ME, Giske CG, Harbarth S, Hindler JF, Kahlmeter G, Olsson-Liljequist B, Paterson DL (2012). Multidrug-resistant, extensively drug-resistant and pandrug-resistant bacteria: an international expert proposal for interim standard definitions for acquired resistance. Clin Microbiol Infect.

[10] Abera B, Kibret M (2011). Bacteriology and antimicrobial susceptibility of otitis media at dessie regional health research laboratory, Ethiopia. Ethiop J Health Dev.

[11] Kazeem MJ, Aiyeleso R (2016). Current bacteriological profile of chronic suppurative otitis media in a tertiary facility of Northern Nigeria. Indian J Otol.

[12] Aduda DS, Macharia IM, Mugwe P, Oburra H, Farragher B, Brabin B, Mackenzie I (2013). Bacteriology of chronic suppurative otitis media (CSOM) in children in Garissa district, Kenya: a point prevalence study. Int J Pediatr Otorhinolaryngol.

[13] Chirwa M, Mulwafu W, Aswani JM, Masinde PW, Mkakosya R, Soko D (2015). Microbiology of chronic suppurative otitis media at queen Elizabeth central hospital, Blantyre, Malawi: a cross-sectional descriptive study. Malawi Med J.

[14] Loy AH, Tan AL, Lu PK (2002). Microbiology of chronic suppurative otitis media in Singapore. Singapore Med J.

[15] Mofatteh MR, Moghaddam FS, Yousefi M, Namaei MH (2018). A study of bacterial pathogens and antibiotic susceptibility patterns in chronic suppurative otitis media. J Laryngol Otol.

[16] Worku M, Bekele M (2014). Bacterial isolate and antibacterial resistance pattern of ear infection among patients attending at Hawassa university referral Hospital, Hawassa, Ethiopia. Indian J Otol.

[17] Hailegiyorgis TT, Sarhie WD, Workie HM (2018). Isolation and antimicrobial drug susceptibility pattern of bacterial pathogens from pediatric patients with otitis media in selected health institutions, Addis Ababa, Ethiopia: a prospective cross-sectional study. BMC Ear Nose Throat Disord.

[18] Muleta D, Gebre-Selassie S (2004). Isolation and antimicrobial susceptibility patterns of bacterial pathogens causing otitis media in children in Jimma Hospital, Southwestern Ethiopia. Ethiop J Health Sci.

[19] Ferede D, Geyid A, Lulseged S, Melaku A (2001). Drug susceptibility pattern of bacterial isolates from children with chronic suppurative otitis media. Ethiop J Health Dev.

[20] Kumar A, Jayachandran L, Kumar S (2016). Antimicrobial susceptibility pattern in chronic suppurative otitis media patient in a tertiary care hospital. Value Health.

[21] Muluye D, Wondimeneh Y, Ferede G, Moges F, Nega T (2013). Bacterial isolates and drug susceptibility patterns of ear discharge from patients with ear infection at Gondar University Hospital, Northwest Ethiopia. BMC Ear Nose Throat Disord.

[22] Melaku A, Lulseged S (1999). Chronic suppurative otitis media in a children’s hospital in Addis Ababa, Ethiopia. Ethiop Med J.

[23] Kumar R, Srivastava P, Sharma M, Rishi S, Nirwan S, Hemwaniand K (2013). Isolation and antimicrobial sensitivity profile of bacterial agents in chronic suppurative otitis media patients at NIMS Hospital, Jaipur. IJPBS.

[24] Adoga AS, Maan EN, Malu D, Badung BP, Obiesie IV, Nwaorgu OG (2010). Swab and aspiration specimen collection methods and antibiogram in chronic suppurative otitis media at Jos University Teaching Hospital: Which is superior?. Ann Afr Med.

[25] Iqbal K, Khan MI, Satti L (2011). Microbiology of chronic suppurative otitis media: experience at Dera Ismail Khan. Gomal J Med Sci..

[26] Pickering LK (2004). Antimicrobial resistance among enteric pathogens. Seminars in pediatric infectious diseases.

[27] Swenson JM, Hindler JA, Peterson LR, Murray PR (1999). Special phenotypic methods for detecting antibacterial resistance. Manual of clinical microbiology.

