# Supplementary material for: Bacterial profile and antimicrobial susceptibility patterns in chronic suppurative otitis media at the University of Gondar Comprehensive Specialized Hospital, Northwest Ethiopia
Source: BMC Res Notes. 2019 Jul 15;12:414. doi: 10.1186/s13104-019-4452-4 (PMC6631645; doi:10.1186/s13104-019-4452-4)
Supplement: Supplementary file 1 — Additional file 1: Table S1. Socio-demographic characteristics of CSOM patients at the University of Gondar Comprehensive Specialized Hospital from January to May 2017. [file 13104_2019_4452_MOESM1_ESM.docx]

Table-S1. Socio-demographic characteristics of CSOM patients at the University of Gondar Comprehensive Specialized Hospital from January - May 2017

| Socio-demographic character | Frequency | Percent |
| --- | --- | --- |
| Sex |  |  |
| Male | 32 | 51.6 |
| Female | 30 | 48.4 |
| Age |  |  |
| < 15 | 20 | 32.3 |
| 16-30 | 27 | 43.5 |
| > 31 | 15 | 24.2 |
| Residence |  |  |
| Rural | 24 | 38.7% |
| Urban | 38 | 61.3% |
| Ear involvement |  |  |
| Right | 32 | 51.6% |
| Left | 26 | 41.9% |
| Both | 4 | 6.5% |
