# Supplementary material for: Bacterial profile and antimicrobial susceptibility patterns in chronic suppurative otitis media at the University of Gondar Comprehensive Specialized Hospital, Northwest Ethiopia
Source: BMC Res Notes. 2019 Jul 15;12:414. doi: 10.1186/s13104-019-4452-4 (PMC6631645; doi:10.1186/s13104-019-4452-4)
Supplement: Supplementary file 2 — Additional file 2: Table S2. Multidrug resistance patterns of the bacterial isolated in CSOM patients at the University of Gondar Comprehensive Specialized Hospital, January to May 2017. [file 13104_2019_4452_MOESM2_ESM.docx]

Table-S2: Multidrug resistance patterns of the bacterial isolated in CSOM patients at the University of Gondar Comprehensive Specialized Hospital, January - May 2017

| Isolates | **Degree of resistance** | | | | | | | | | |
| --- | --- | --- | --- | --- | --- | --- | --- | --- | --- | --- |
|  | R0  n (%) | R1  n (%) | R2  n (%) | R3  n (%) | R4  n (%) | R5  n (%) | R6  n (%) | R7  n (%) | R≥8  n (%) | MDR****** |
| *S. aureus (n=12)* |  | 4(33.3) | 3(25) | 1(8.3) |  | 1(8.4) | 2(16.6) |  | 1(8.4) | 5(41.7) |
| *P. vulgaris (n=2)* |  |  |  | 1(50) |  |  |  |  | 1(50) | 2(100) |
| *Enterobacter* spp*(n=4)* |  | 4(100) |  |  |  |  |  |  |  | 0 |
| *CoNS****** *(n=12)* |  | 3(25) | 4(33.3) | 2(16.6) | 1(8.3) |  | 1(8.4) | 1(8.4) |  | 5(41.7) |
| *Pseudomonas* spp*(n=2)* |  |  |  |  | 1(50) |  | 1(50) |  |  | 2(100) |
| *Providencia* spp*(n=11)* |  | 1(9.1) |  | 1(9.1) | 0 |  | 2(18.2) | 1(9.1) | 6(54.6) | 10(90.9) |
| *P. mirabilis*  *(n=16)* |  | 4(25) | 5(31.1) | 1(6.3) | 4(25) | 1(6.3) | 1(6.3) |  |  | 7(43.8) |
| *Citrobacter* spp*(n=5)* |  | 3(60) |  | 1(20) |  | 1(20) |  |  |  | 2(40) |
| *Klebsiella* spp*(n=10)* | 1(10.0) | 1(10) | 6(60) |  | 2(20) |  |  |  |  | 2(20) |
| Total*(n=74)* | 1(1.4) | 20(27) | 18(24.3) | 7(9.4) | 8(10.8) | 3(4.1) | 7(9.5) | 2(2.7) | 8(10.8) | 35(47.3) |

*CoNS*= Coagulase negative Staphylococci, **MDR=Isolates resistant to 3 or more antibiotics classes. R0= All are sensitive, R1= Resistant to one antibiotic classes, R2= Resistant to two antibiotic classes etc.., R>8= Resistant to greater or equal to 8 antibiotics*
